# Clinical and pathological findings in two Italian siblings of Romani ancestry with charcot-marie-tooth type 4D and review of the current literature

**DOI:** 10.1177/22143602261438536

**Published:** 2026-05-06

**Authors:** Elena Abati, Carola Rita Ferrari Aggradi, Stefania Magri, Chiara Pisciotta, Francesca Balistreri, Giacomo Comi, Davide Pareyson, Franco Taroni, Stefania Corti

**Affiliations:** 1 Department of Pathophysiology and Transplantation (DEPT), 9339 University of Milan, Milan, Italy; 2 Neurology Unit, Fondazione IRCCS Ca’ Granda Ospedale Maggiore Policlinico, Milan, Italy; 3 Unit of Medical Genetics and Neurogenetics, 9328 Fondazione IRCCS Istituto Neurologico Carlo Besta, Milan, Italy; 4 Unit of Rare Neurological Diseases, Department of Clinical Neurosciences, 9328 Fondazione IRCCS Istituto Neurologico Carlo Besta, Milan, Italy; 5 Neuromuscular and Rare Diseases Unit, Fondazione IRCCS Ca’ Granda Ospedale Maggiore Policlinico, Milan, Italy

**Keywords:** CMT4D, CMT-Lom, NDRG1, n-myc downstream-regulated gene 1, hereditary neuropathy

## Abstract

Charcot-Marie-tooth disease type 4D (CMT4D) is an early-onset, severe autosomal recessive demyelinating neuropathy, caused by mutations in the N-myc downstream-regulated gene 1 (*NDRG1*). Because of its rarity and predominance among specific ethnic groups, clinical knowledge remains limited. We report the case of two siblings of Romani ancestry, a 38-year-old man and a 40-year-old woman, with homozygous NM_006096.4:c.442C > T, p.(Arg148*) variant, and provide a review of the current literature.

Both patients presented with severe distal-proximal sensorimotor neuropathy, muscle weakness and atrophy, generalized areflexia, and sensorineural deafness typical of CMT4D. Notably, they exhibited previously unreported features including severe dysphagia requiring PEG tube placement, bilateral vocal cord paralysis causing respiratory insufficiency necessitating non-invasive ventilation, and cognitive delay. Visual system involvement was demonstrated through abnormal visual evoked potentials with reduced P100 amplitude and absent responses, expanding the recognized phenotypic spectrum.

Our systematic literature review identified 26 articles describing 72 patients with CMT4D. Twenty-two pathogenic *NDRG1* variants were documented, with p.(Arg148*) being most frequent (64% of patients), predominantly in Roma populations. The median age of onset was 7 years, 96% of patients presented with lower limb involvement and all presented skeletal deformities were universal, including pes cavus (67%), claw hand (40%), and scoliosis (33%). Hearing impairment affected 61% of patients, while visual system involvement occurred in 17%.

This study expands the clinical spectrum of CMT4D by documenting novel manifestations including severe bulbar dysfunction and respiratory involvement. These findings emphasize the importance of comprehensive assessment including swallowing evaluation, vocal cord examination, and pulmonary function testing in CMT4D patients, potentially impacting clinical management and prognosis.

## Introduction

**Figure 1.**
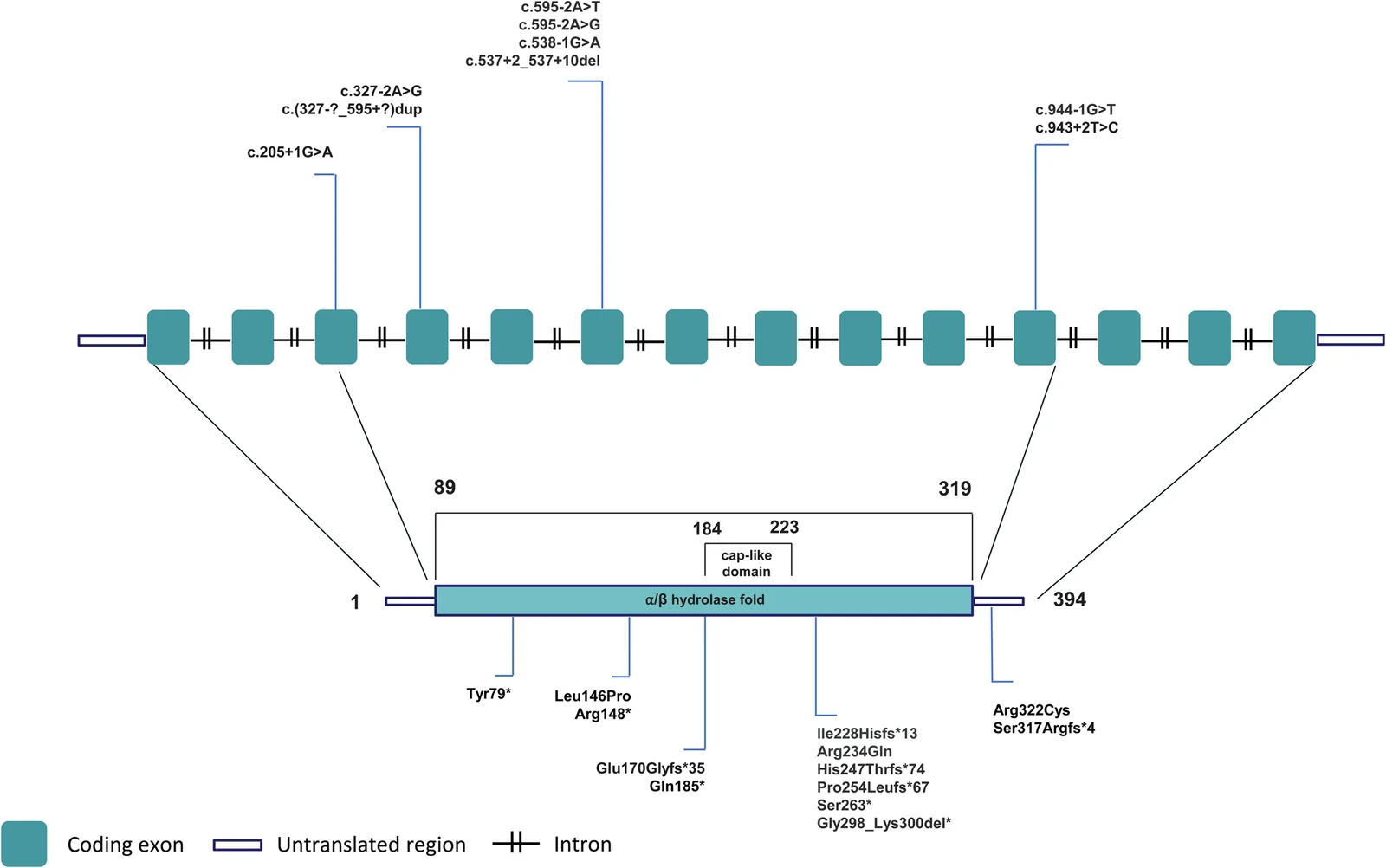
Schematic view of NDRG1 transcript and protein reporting the localization of point mutations and small indels detected in CMT4D patients.

Charcot-Marie Tooth disease (CMT) is the most common inherited neuromuscular disorder, with a prevalence of 1 in 2500 people.^1^ It mainly manifests as predominantly distal sensorimotor polyneuropathy, but highly heterogeneous genetic and clinical variants are reported.^2^ Among CMT subtypes, CMT type 4 (CMT4) traditionally refers to autosomal recessive, demyelinating forms. *NDRG1*-associated neuropathy, also known as CMT4D), is a very rare autosomal recessive disease characterized by severe and early-onset demyelinating sensorimotor polyneuropathy leading to generalized areflexia, muscle weakness and atrophy, skeletal deformities such as pes cavus, scoliosis, and claw hand, walking difficulty, and sensorineural deafness.^3,4^ It was first described in families of Romani ancestry in the region of Lom in 1996, and referred to as hereditary motor and sensory neuropathy Lom (HMSN-L).^4^ The first identified mutation was the NM_006096.4:c.442C > T p.(Arg148din) in the N-myc downstream regulated gene 1 (*NDRG1*), located on chromosome 8q24.3 and encoding a 43 kDa protein.^4,5^ NDRG1 protein contains a central globular α/β hydrolase fold, an N-terminal 30-residue region and a C-terminal region containing three 10-residue repeats (Figure 1).^6^ NDRG1 is thought to play a role in cell growth and differentiation and is ubiquitously present in human tissues and highly expressed in the peripheral nervous system.^7^ A study showed that the sciatic nerve of *Ndrg1*-deficient mice underwent a demyelinating process a few weeks after birth, suggesting a key role of NDRG1 in maintaining myelin sheets in peripheral nerves.^8^ Twenty-two different *NDRG1* pathogenic variants, among point mutations, small indels and copy number variations, have been identified so far (Table 1).^5,9–25^ Figure 1 details the localization of point mutations and small indels along the transcript and protein.

**Table 1.** Known pathogenic mutations within the NDRG1 gene. All variants are annotated using NM_006096.4 as the reference transcript. Intronic variants that affect splicing and don't have direct protein-level predictions are indicated by “-”.

Genomic position	Nucleotide change	Amino acid change	Reported in	Phenotype
chr8:134251342	NM_006096.4:c.964C > T	p.(Arg322Cys)	Yalcintepe et al.2021	CMT
chr8:134251358	NM_006096.4:c.948del	p.(Ser317Argfs*4)	Dohrn et al.2017	CMT
chr8:134251363	NM_006096.4:c.944-1G > T	-	Alfares et al.2017	CMT
chr8:134254264	NM_006096.4:c.943 + 2T > C	-	Uchôa Cavalcanti et al.2021	Early-onset CMT with scoliosis, vocal cord palsy, restrictive lung disease
chr8:134254318	NM_006096.4:c.892-1G > T	p.(Gly298_Lys300del)*	Cortese et al.2020	Mild CMT without hearing and eye impairment
chr8:134260137	NM_006096.4:c.788C > A	p.(Ser263*)	Uchôa Cavalcanti et al.2021	CMT
chr8:134280394	NM_006096.4:c.761del	p.(Pro254Leufs*67)	Monies et al.2017	CMT with facial and lower limb weakness, abnormal proprioception, sensory ataxia, pes cavus and hearing impairment
chr8:134260974	NM_006096.4:c.739del	p.(His247Thrfs*74)	Piscosquito et al.2017	CMT with pes cavus and hearing impairment
chr8:134261012	NM_006096.4:c.701G > A	p.(Arg234Gln)	Li et al.2017	CMT with pes cavus and hearing impairment
chr8:133250457	NM_006096.4:c.681dup	p.(Ile228Hisfs*13)	Volodarsky et al.2021	CMT
chr8:134262788	NM_006096.4:c.595-2A > G	-	Chen et al.2019	Early-onset CMT with distal lower limbs involvement, no hearing nor eye involvement
chr8:134262788	NM_006096.4:c.595-2A > T	-	Akbar et al.2023	N/A
chr8:134266823	NM_006096.4:c.553C > T	p.(Gln185*)	Chen et al.2018	Early-onset CMT with pes cavus, scoliosis, claw hands, hearing impairment, hand tremor and tongue atrophy
chr8:134266839	NM_006096.4:c.538-1G > A	-	Hunter et al.2003	CMT with pes cavus, scoliosis, hearing impairment
			Atkinson et al. 2024	Early-onset CMT with moderate-severe weakness
chr8:134269010	NM_006096.4:c.537 + 2_537 + 10del	-	Pravinbabu et al., 2022	CMT with pes cavus, hearing impairment, prolonged P100 latencies, subcortical white matter T2 hyperintensities at brain MRI
chr8:134270555	NM_006096.4:c.508dup	p.(Glu170Glyfs*35)	Atkinson et al. 2024	Early-onset CMT with moderate-severe weakness
chr8:134270617	NM_006096.4:c.442C > T	p.(Arg148*)	Kalaydjieva et al.2000; Also described in: Luigetti et al, 2014; Ricard et al, 2013; Dackovic et al, 2008; Echaniz-Laguna et al, 2007; Parman et al, 2004.	Multiple CMT patients with pes cavus (19/39), claw hand (17/39), scoliosis (8/39), hearing impairment (25/39), Horizontal nystagmus (2), impaired light reflex (2), tongue atrophy (9/39)
chr8:134270622	NM_006096.4:c.437T > C	p.(Leu146Pro)	Li et al.2017	CMT with pes cavus and dysartrhia
chr8: 134265065-134271319	NM_006096.4:c.(327-?_595+?)dup	-	Okamoto et al., 2014	Early-onset CMT with pes cavus, scoliosis, claw hand, and hearing impairment
chr8:134276767	NM_006096.4:c.327-2A > G	-	Atkinson et al. 2024	Early-onset CMT with moderate-severe weakness
chr8:134274379	NM_006096.4:c.237C > A	p.(Tyr79*)	Candayan et al.2021	N/A
chr8:134276789	NM_006096.4:c.205 + 1G > A	-	Yalcintepe et al.2021	CMT

*NDRG1*-associated neuropathy is extremely rare and typically found in a historically disadvantaged ethnic group. These two factors contribute to making it poorly known among clinicians and result in a scarcity of related literature. After encountering an affected family with symptoms that have never been reported previously, we felt the need to gather available data and provide fellow clinicians with an updated review of the *NDRG1*-associated spectrum. In this study we thus report the case of two Italian siblings of Romani ancestry carrying the homozygous *NDRG1* pathogenic variant p.(Arg148*), expanding the spectrum of its manifestations, and provide a review of available literature on the genetic and clinical features of patients affected by CMT4D.

## Materials and methods

### Patients’ clinical and instrumental evaluation

Two siblings, a 40-year-old woman and a 38-year-old man, affected by CMT4D with confirmed homozygous NM_006096.4:c.442C > T p.(Arg148*) variant were admitted to the Inpatient Clinic of the Department of Neurology at the IRCCS Fondazione Ca’ Granda Ospedale Maggiore Policlinico in 2022. Neurologists experienced in neuromuscular diseases performed neurological clinical examinations and chart reviews at admission. The patients underwent a series of instrumental evaluations, including blood tests, nerve conduction studies (NCS), electromyography (EMG), visual evoked potentials (VEP), eye examination, brain magnetic resonance imaging (MRI), spirometry, nocturnal pulse oximetry pneumological examination, ear, nose and throat (ENT) examination, and fiberoptic endoscopic evaluation of swallowing (FEES). Charcot–Marie–Tooth Examination Score version 2 (CMTESv2), a subscore of Charcot–Marie–Tooth Neuropathy Score version 2 (CMTNSv2), and its Rasch-weighted version (CMTNSv2-R), were used to estimate disease severity and clinical disability in the patients.^26–29^

### Review of the literature: article selection

Articles were included if they met the following criteria: original articles, case reports or case series, published in peer-reviewed journal articles which included one or more participant clinically or genetically diagnosed with CMT4D Article search was performed in PubMed using the following keywords: “CMT4D”, “CMT4”, “Charcot-Marie-Tooth Type 4D”, “Charcot-Marie-Tooth Type 4”, “Hereditary Motor and Sensory Neuropathy-Lom”, “HMSNL” or “Charcot-Marie-Tooth” and the additional keyword “*NDRG1*” or “*NDRG1* mutation”. No time interval was set as a filter. The search was carried out in April 2025, yielding 26 papers . Titles, abstracts, and entire tex twere reviewed. Additionally, we searched the Human Gene Mutation Database (HGMD^®^ Pro version, https://www.hgmd.cf.ac.uk) for published variants in the *NDRG1* gene. Clinical, electrophysiological, and genetic data from the selected articles were then recorded.

### Statistical analysis

Baseline characteristics were analysed through descriptive statistics, continuous variables were reported as mean ± SD or median [IQR], as appropriate. Categorical variables were represented as numbers and percentages. Fisher's exact test was performed to evaluate the association between mutation type (truncating vs. non truncating) and clinical parameters. Statistically significant differences were assumed at 5% level of probability, and all statistical tests were two-tailed. All analyses were performed with GraphPad Prism version 10 (GraphPad Software Inc)**.**

## Results

## 1. Patients’ genetic and clinical characteristics

Patient 1 was a 40-year-old female who previously received a genetically determined diagnosis of CMT4D with the known homozygous *NDRG1* pathogenic variant NM_006096.4:c.442C > T p.(Arg148*). Patient 2 was her 38-year-old brother who received the same genetic diagnosis.

Patient 1 started walking at the age of 1 year, but with immediate gait impairment and predominantly on toes. Later, when she was 5-year-old, also the upper limbs were involved, with fine hand movement impairment. At the age of 5, she underwent a bilateral pes cavus surgical correction. She was able to maintain unassisted walking until the age of 20, then she started using a cane. At the age of 27 she moved on to using a walker, and she became wheelchair dependent at the age of 36. Dysphagia began at the age of 18, leading to three episodes of aspiration pneumonia at 33, 36, and 38 years of age. At age of 36, she was diagnosed with bilateral vocal fold paralysis in adduction causing dyspnea. Deafness started in childhood. The patient had a history of cognitive delay since childhood, although this was not formally assessed.

Previous medical records were not available for Patient 2, and clinical history was difficult to reconstruct because of the partial language barrier and lack of compliance. He had signs of bilateral malleolar surgical stabilization, and a known history of hearing loss, breathing impairment and cognitive delay.

Their parents were reported healthy, both of Romani ancestry but apparently nonconsanguineous. The patients had three other sisters, two reported to have a clinical picture compatible with CMT and one healthy. One of the affected sisters had died at the age of 36 because of the complications of a tracheotomy, the other one was 31-year-old and still able to walk independently. Segregation studies could be performed only in two sisters, one affected, who tested positive for the same variant, and one healthy, who tested negative. It was not possible to review them clinically. Samples from other family members were not available.

Upon admission, patient 1's neurological examination revealed severe hearing loss, dysarthric and hypophonic speech, reduced visual acuity, kinetic head tremor, mild face and jaw weakness, generalized areflexia and severe limb muscle atrophy weakness, with a disto-proximal gradient. Specifically, muscle strength was assessed with the Medical Research Council (MRC) scale, showing severe distal weakness and moderate-to-severe proximal weakness in both upper and lower limbs. Detailed MRC scores are provided in Table 2. In addition, she had reduced pinprick sensibility in the feet (up to the ankle) and ankle and tibial crest hypopallesthesia. She was not able to stand on her own. The patient did not present scoliosis at physical examination, not it was present at chest X-ray scans.

**Table 2.** Medical research council (MRC) scale for muscle strength scores in patients 1 and 2.

Muscle groups	Patient 1		Patient 2	
	*Right*	*Left*	*Right*	*Left*
*Upper limbs*				
Deltoids	5	5	5	5
Elbow flexors	3	3	4	4
Elbow extensors	3	3	4	4
Wrist flexors	2	2	3	3
Wrist extensors	2	2	3	3
Finger flexors	1	1	1	1
Finger extensors	1	1	1	1
Palmar interosseus	1	1	1	1
*Lower limbs*				
Hip flexors	4	4	5	5
Knee flexors	2	2	4	4
Knee extensors	2	2	4	4
Feet dorsiflexors	1	1	1	1
Feet plantar flexors	1	1	1	1
Hallux extensors	1	1	1	1

Patient 2's neurological examination showed severe hearing loss, dysarthric speech, reduced visual acuity, torsional nystagmus, jaw weakness, generalized areflexia, severe limb muscle atrophy and weakness, predominantly distal, bilateral Achilles tendon retraction, and bilaterally reduced pinprick sensibility in a glove-and-stocking distribution. Muscle strength assessment revealed severe distal weakness and mild proximal weakness in both upper and lower limbs (Table 2). He was able to reach and maintain the standing position independently but could walk only if using two canes. The patient did not present scoliosis at physical examination, not it was present at chest X-ray scans.

**Patient 1.** Routine blood tests, including complete blood count, kidney and liver function, electrolytes, iron studies, folate and B12 vitamin, creatine kinase (CK) and protein electrophoresis, were normal except for CK (22 U/l; normal range 30–150 U/l) and iron (26 mcg/dl; normal range 50–150 mcg/dl).

Electrophysiological examination showed absent sensory nerve action potential (SNAP) and compound muscle action potential (CMAP) in the right radial, median, tibial, and ulnar nerves. NCS of the axillary nerve and the facial nerve showed prolonged distal latencies with decreased CMAP amplitude (Table 3). Needle EMG of the deltoid muscle displayed denervating potentials, namely fibrillation potentials and positive sharp waves, and motor unit potentials (MUP) typical of chronic neuropathic damage, with increased amplitude and duration and reduced recruitment. Conversely, while needle EMG of the frontalis muscle displayed severe signs of chronic neuropathic damage with significant reinnervation, in absence of any active or uncompensated denervation signs in this more proximal muscle. The Charcot-Marie-Tooth Disease Neuropathy Score - version 2 (CMTNSv2) was 31/36, while Rasch-weighted score was 36/40.

**Table 3.** Motor and sensory nerve conductions studies (NCS). The table shows patient 1 (I) and 2 (II) electrophysiological exams. Distal nerve CMAPs and SAPs were not elicitable. Proximal and cranial nerve studies revealed severely reduced conduction anomalies of demyelinating type.

Motor conduction studies	I	II
*Right posterior tibial nerve*	NE	NE
*Right median nerve*	NE	NP
*Right ulnar nerve*	NE	NE
*Right axillary nerve*		
Latency (deltoid)	19.0 ms	13.6 ms
Amplitude (deltoid)	0.2 mV	0.9 mV
*Right facial nerve*		
Latency (orbicularis oculi)	11.4 ms	
Amplitude (orbicularis oculi)	0.1 mV	
Latency (frontalis)		9.8 ms
Amplitude (frontalis)		0.2 mV
Sensory conduction studies	I	II
*Right sural nerve*	NE	NE
*Right radial nerve*	NP	NE

Normal values. Axillary nerve: distal latency < 4.9 ms; amplitude > 3.5 mV; Facial nerve (orbicularis oculi): amplitude > 1 mV; latency < 4.2 ms. Facial nerve (frontalis): amplitude > 2 mV; latency < 2.1 ms.

NE = Not elicitable; NP = Not performed.

VEP displayed a P100 of low amplitude with normal latency at 60’ stimulation, and an absent P100 response at the 15’ stimulation. Ophtalmoscopic evaluation showed visual acuity of 6–7/10 in both eyes, while evaluation of the fundus oculi showed a pale optic disc with mild angiosclerosis.

Brain MRI was normal.

The ENT visit, comprehensive of FEES, confirmed the presence of a bilateral vocal fold palsy in adduction and showed an absent cough reflex and severe dysphagia for both solid and liquid foods that required the placement of a Percutaneous Endoscopic Gastrostomy (PEG) tube. Nocturnal pulse oximetry was normal (mean SpO2 97.4%, T SpO2 < 90% 2.0%, average of minimum SpO2 levels 87.9%, ODI index 0.4). Spirometry showed a reduction of Forced Vital Capacity (FVC) to 44% of predicted, a reduction of Forced Expiratory Volume in one second (FEV1) to 30% of predicted, a reduction of Vital Capacity (VC) both in the supine and standing position (0.54 L (24% predicted) and 0.67 L (30% predicted) respectively, and reduction in the Peak Cough Flow (PCF) (1.58 L/sec (29% predicted), while Maximal Inspiratory Pressure (MIP) and Maximal Expiratory Pressure (MEP) could not be measured due to severe reduction in strength below the detection capability of the device. The patient was then diagnosed with a severe restrictive respiratory syndrome causing a chronic respiratory insufficiency with cough impairment. The pneumologist suggested non-invasive ventilation (NIV) and cough assist adaptation.

**Patient 2.** Routine blood tests were normal except for CK (22 U/l; normal range 30–200 U/l) and iron (45 mcg/dl; normal range 50–150 mcg/dl). Electrophysiological examination showed absent CMAP in the tibial and ulnar nerves and absent SNAP in the radial and sural nerves. NCS of the axillary nerve and the facial nerve showed prolonged distal latencies with decreased CMAP (Table 2). Needle EMG of the vastus medialis displayed fibrillation potentials, while no voluntary activity could be elicited. Needle EMG of the deltoid, the biceps brachii, and the frontalis muscles displayed severe/moderate signs of chronic neuropathic damage, including increased MUP amplitude and duration and reduced recruitment, without spontaneous activity. CMTNSv2 was 32/36 and CMTNS-R 34/40.

VEP displayed a P100 of low amplitude with normal latency at 60’ stimulation, and an absent P100 response at the 15’ stimulation. Ophtalmoscopic evaluation showed visual acuity of 9/10 in both eyes, while evaluation of fundus oculi was normal.

The patient refused to undergo brain MRI.

The ENT visit, comprehensive of FEES, showed mild dysphagia for solid foods, and recommended transition to a soft diet.

Nocturnal pulse oximetry showed the presence of some periods with initial phasic/tonic nocturnal desaturations with SpO2 values that remain within normal limits (mean SpO2 95.3%, T SpO2 < 90% 0.3%, average of minimum SpO2 levels 90.8%, ODI index 4.1). Spirometry showed borderline FVC and FEV1 (respectively 70% and 75% of predicted), reduced VC in the supine position and normal VC in the standing position (3.01 L (72% predicted) and 3.54 L (85% predicted) respectively), normal PCF (8.61 L/sec (102% predicted)) and reduced MIP and MEP (54 cmH2O (52% predicted) and 57 cmH2O (40% predicted) respectively). The results suggested mild hypoventilation and an initial loss of respiratory muscle strength, needing regular clinical and instrumental follow up.

## 2. Review of the current literature

Twenty-six articles, all published in English between 1996 and 2025, met the criteria used for paper selection.^4,5,9–25^^,30–37^ All patients’ clinical, electrophysiological and additional details are available in Supplementary Table 1.

### 2.1 Demographic characteristics

The number of CMT4D patients included in the articles ranged from 1 to 39, with a median of 1 patient (Supplementary Table 1). Clinical and demographic data were not available for 7 patients. Patients’ age at hospital admission ranged from 13 to 43, with a median age of 22 (mean = 22.6), and median age at onset ranged from 5 to 12 years, with a median age of 7 (mean = 7.2). Overall, out of 72 aggregated patients, both genders were relatively equally represented, with similar proportions of male and female patients. Fifty-four (75%) patients were of Romani ancestry. The 18 non-Romani patients were from Bulgaria (n = 9), China (n = 3), Turkey (n = 3), Italy (n = 2), and India (n = 1).

### 2.2 Genetic characteristics

Four papers reported non-specific alteration in a locus on chromosome 8q24.^4,30,31,37^ The remaining 22 papers described a total of twenty-two pathogenic variants in *NDRG1*. The most frequent variant was NM_006096.4:c.442C > T p.(Arg148*), detected in 46 (64%) patients, all of Romani ancestry.^5,32–36^ In order of decreasing frequency, the other most commonly reported variants were c.538–1G > A (n = 6),^14,25^ c.327–2A > G (n = 4),^25^ exon 6–8 duplication (n = 3).^18^ The remaining variants were described in one patient each. The pathogenic variant was not available for four patients reported prior to the discovery of the disease gene.^30,31^ However, these patients carried the same 8q24 haplotype observed in the original HMSN-Lom families,^4,5^ making them likely carriers of common p.(Arg148*) *NDRG1* mutation.

### 2.3 Clinical characteristics

Out of 70 patients for whom this information was reported, the vast majority (n = 67, 96%) exhibited lower limb involvement, with leg weakness and walking difficulties, as the first symptom of the disease (Table 4). Two patients had delayed motor milestones, and only one complained of hand weakness as the first symptom. At their first neurological evaluation, all patients had walking difficulties, distal upper and lower limb muscle weakness and hypo-atrophy, generalized areflexia, and skeletal deformities. Among skeletal deformities, pes cavus was found in 47/70 patients (67%), claw hand in 28/70 (40%), and scoliosis in 23/70 (33%). Tongue atrophy was present in 10 (14%) patients. Hand tremor was reported in three patients (3/70, 4.29%). We reported dysarthria in our two patients, that was reported previously only in three other patients (5/70, 7.14%).^19,34^ Dysphonia was reported in patient 1 in this case and in another patient (2/70, 2.86%).^16^ Our patients also presented moderate/severe dysphagia that has never been reported in the literature previously (2/70, 2.86%). Hearing impairment was found in 43/70 (61%) patients, with the notable observation that some Bulgarian patients had normal audiograms up to the fourth decade despite subclinical auditory pathway involvement on BAEP.^25^ Visual system involvement, including impaired light reflex, reduced visual acuity, myopia and glaucoma, was reported in 9, out of 53 for whom the information was provided (17%). Alterations of fundus oculi (pale disc) or retinal thinning at ocular computer tomography (OCT) are reported in 3 patients.^16,18,30,33,37^VEPs were performed in one patient and found to be abnormal with prolonged P100 latencies, while our patients both displayed absence of P100 wave at 15’ and reduced amplitude of P100 at 60’^20^ . Nystagmus was reported in 6 patients, including patient 2 described in this paper.^16,30,33,37^ Our patients both presented restrictive pulmonary disease, more severe in patient 1, who also had bilateral vocal cord palsy and hypophonia. Previously, vocal cord involvement was described in only one patient, who also had restrictive pulmonary disease at spirometry.^21^ Data regarding respiratory function have not been provided for any other patient, and respiratory involvement was not reported. As regards central nervous system involvement, MRI results were reported for 9 patients, yielding normal scans in 5 cases. In the remaining cases, hyperintense T2 lesions in the subcortical white matter were found in two cases,^20,33^ focal lesions in the nucleus caudatus and parietal lobe were observed in one case,^36^ and mild cerebellar atrophy and slight thinning of the medulla oblongata were observed in one case.^16^ Our patients both had a history of cognitive delay, that was not reported in any other study in the literature.

**Table 4.** Clinical presentation of NDRG1-associated neuropathy. Number of patients reporting each symptom or sign are given, as well as percentages on the total number for whom the information was available (the total is 70 patients unless otherwise specified). * = Data regarding results of these procedures was only reported for 6 patients in the case of fundus oculi and OCT, and for 3 patients in the case of VEPs, therefore percentages are not given.

Initial presentation	Number of patients (percentage)
Age of onset (*median, IQR*)	6.5 (4.50–7.25)
Lower limb involvement	67 (96%)
Hand weakness	1 (2.86%)
Delayed motor milestones	2 (1.43%)
*Clinical picture*	
Skeletal deformities	70 (100%)
Scoliosis	23 (33%)
Pes cavus	47 (67%)
Claw hand	28 (40%)
Tongue atrophy	10 (14%)
Hand tremor	3 (4.29%)
Dysarthria	5 (7.14%)
Dysphonia	2 (2.86%)
Dysphagia	2 (2.86%)
Hearing impairment	43 (61%)
Visual system involvement (impaired light reflex, reduced visual acuity, myopia and/or glaucoma)	9/53 (17%)
Alterations of fundus oculi/abnormal OCT*	3/6
Altered VEPs*	3/3
Nystagmus	6 (8.57%)
Vocal cord palsy	2 (2.86%)
Restrictive pulmonary disease	3 (4.29%)
Cognitive delay	2 (2.86%)

### 2.4 Electromyoneurographic characteristics

Exhaustive electromyoneurographic (EMNG) assessment was available for 30 (41%) patients. All these patients had a severe distal demyelinating sensorimotor polyneuropathy, with reported secondary axonal damage and (active/chronic) denervation in 22 cases (73%). The study by Atkinson et al.^25^ confirmed deterioration of nerve conduction parameters over time in patients with longitudinal follow-up. Median or ulnar nerve motor conduction velocity parameters were available for 68 patients. In 17 patients, CMAP was not elicitable. In the remaining patients, mean values for median CMAP conduction velocity are 13.24 ± 1.39 (SEM).

### 2.5 Genotype-phenotype correlation

The majority of patients display a moderate/severe phenotype with early onset, skeletal deformities and hearing loss. Other additional symptoms are reported in smaller subsets of patients. We attempted to verify whether truncating mutations could result in a more severe phenotype and in a higher rate of CNS signs or bulbar involvement. We analysed the association between mutation status (truncating vs. non truncating) and presence of CNS signs (defined as clinical or instrumental visual involvement, cognitive delay and/or presence of abnormalities at MRI scans) or bulbar involvement (vocal cord palsy, dysphagia, and/or dysarthria), but no statistically significant correlation could be observed.

## Discussion

The findings from our study contribute to the knowledge on CMT4D, a rare and severe form of inherited neuromuscular disorder characterized by sensorimotor polyneuropathy. CMT4D is a recessive disease, and because of its rarity and recurrence mainly in ethnic minorities, there are still relatively few cases reported in the literature. By reporting the case of two Italian siblings of Romani ancestry with a known mutation in *NDRG1,* p.(Arg148*), and reviewing current literature, our study summarizes its clinical and genetic spectrum and expands the spectrum of known clinical manifestations of this disease.

CMT4D typically presents with early-onset demyelinating sensorimotor neuropathy, leading to distal weakness that extends proximally as the disease progresses, distal sensory loss, foot deformities, and sensorineural hearing loss. The identification of the p.(Arg148*) mutation in our patients is consistent with previous findings that this variant is the most frequent pathogenic mutation causing CMT4D, predominantly found in patients of Romani descent. This mutation was initially identified in the Roma population in the Lom region of Bulgaria^4^ and subsequently recognized in Roma communities across Europe.^5^

Our patients displayed a severe phenotype, characterized by early onset, severe motor and sensory involvement in both upper and lower limbs, pes cavus, and severe demyelinating neuropathy at conduction studies. The early age of onset (first decade of life) and the clinical manifestations align with the expected severe phenotype associated with the p.(Arg148*) variant. Our patients also presented evidence of CNS involvement, such as sensorineural deafness, which is a common report in patients with *NDRG1*-associated neuropathy (Table 4), and reduced visual acuity with instrumental evidence of optic nerve dysfunction at VEPs. Interestingly, only one previous case was reported to have abnormal VEP responses^20^ . MRI scans in patient 1 were normal, but there are reports in the literature of abnormal MRI findings such as hyperintense T2 lesions in the subcortical white matter and deep nuclei, and atrophy of cerebellum and medulla oblungata. Our patients also presented other uncommon symptoms, such as dysarthria, dysphonia and severe dysphagia. As regards the latter, this is so far the first report of dysphagia in CMT4D patients. Another notable clinical feature is the bilateral vocal cord paresis displayed by patient 1 and the severe respiratory insufficiency requiring the use of NIV. A milder respiratory syndrome was present also in patient 2, causing mild hypoventilation and an initial loss of respiratory muscle strength. Previously, only one CMT4D patient was reported to have vocal cord palsy and restrictive pulmonary disease at spirometry.^21^ Vocal cord palsy was described in other CMT subtypes, e.g., *GDAP1*-associated neuropathy and *MFN2*-associated neuropathy, especially in patients with severe phenotype including upper limb weakness, respiratory involvement and other cranial nerve dysfunctions.^38,39^ Sevilla and colleagues performed neurophysiological evaluations on eight patients with vocal cord palsy, confirming phrenic nerve dysfunction.^38^ All the laryngeal muscles except for the cricothyroid muscle are innervated by the recurrent laryngeal nerve, a branch of the vagus. Paralysis in adduction is probably due to the tensor action of the cricothyroid muscle which is innervated by the superior laryngeal nerve, also a branch of the vagus but shorter than the recurrent nerve, and it is usually spared or less affected. Sevilla and colleagues hypothesize that paresis of the vocal cords could represent a stage in the neuropathy and may be an indicative parameter of the severity of the disease. Dysphagia is probably due to dysfunction of the pharyngeal branches of the vagus nerve, whose path is shorter than laryngeal recurrent nerves. Our patients also presented respiratory dysfunction. Patient 1 had severe respiratory insufficiency with hypoventilation, severe reduction of global respiratory muscle strength (as measured by MIP and MEP) and cough impairment requiring the use of NIV, while patient 2 presented milder respiratory symptoms. Respiratory complications are a major cause of morbidity and mortality in neuromuscular diseases and can lead to aspiration pneumonia, fatal respiratory insufficiency or to sudden death during sleep. The concomitant presence of vocal cord paresis may increase the likelihood of such events. It is therefore important to specifically investigate patients with CMT due to *NDRG1* mutations for evidence of laryngeal and respiratory dysfunction. Overall, our findings show that patients with *NDRG1-*related neuropathy have a length-dependent neuropathy that, while affecting more distal muscles of the limbs initially, may extend to the cranial nerves and respiratory muscles^4,20,33,35^

Our literature review identified twenty-two different pathogenic variants in *NDRG1* reported to date, with the p.(Arg148*) variant being the most common. Notably, most reported pathogenic variants in *NDRG1* are truncating mutations (nonsense, frameshift, or splice-site mutations), with only a few missense mutations described. In physiological conditions, NDRG1 protein is highly expressed in the central and peripheral nervous system, where it is enriched in the cytoplasm of myelinating cells.^5,40^ The predominance of truncating mutations suggests a loss-of-function mechanism in CMT4D pathogenesis, as supported by studies in *NDRG1*-deficient mice showing peripheral demyelination starting at 5 weeks of age.^8,41^ Interestingly, myelin was normal until the onset of demyelination, which eventually resulted in axonal damage, especially of large caliber axons. It was therefore suggested that NDRG1 is crucial for peripheral nerve health by regulating trafficking and transport within the cytoplasm of Schwann cells, to meet the increasing demands of growing nerves. Marechal and colleagues generated a lineage specific conditional knockout (*Olig1^cre/+^;Ndrg1 ^fl/fl^*) line to study central myelination, and reported defective maintenance of mature oligodendrocytes and myelin regenerative potential in such line. A potential explanation was given by previous studies in immortalized oligodendrocyte progenitor cell lines, which proposed a role for NDRG1 in lipid homeostasis and subcellular transport.^41,42^ The potential effects of NDRG1 on CNS myelination are reflected by the presence of CNS signs in a variety of published CMT4D patients, including 4 patients with MRI abnormalities, mainly white matter alterations,^16,20,33,36^ and 17% of the patients reported in our literature review showing clinical or instrumental visual system impairment.^16,20,30,33,37^ The two patients that we describe also showed evidence of VEP alterations and cognitive delay. A recent study also highlighted the role of NDRG1 in iron metabolism and ferroptosis in cardiomyocytes, with iron overload, elevated levels of reactive oxygen species (ROS) and lipid peroxidation in NDRG1-deficient cardiomyocytes.^43^ NDRG1 seems to interact with transferrin and to promote transferrin recycling by interacting with cell membrane.^43^ The effects of NDRG1 on iron metabolism in the serum or in other cell types are currently unknown, but interestingly both our patients presented reduced iron levels with normal transferrin levels in the blood. These findings could be due to poor nutritional intake but as of now we cannot exclude it could be associated, at least in part, with *NDRG1*-related disease. Overall, NDRG1 effects on lipid and iron metabolism seem to be linked to vesicle-mediated transport processes and protein sorting.^41–43^

As regards genotype–phenotype correlation, the small number of patients does not allow to make precise inferences.As pointed out above, the majority of patients display a moderate/severe phenotype with early onset, skeletal deformities and hearing loss. No association was retrieved between the presence of other addition symptoms (CNS signs, bulbar involvement) and mutation status (truncating vs. non-truncating). Further studies on larger series of patients would be useful in order to clarify the potential presence of genotype-phenotype correlations.

From a clinical perspective, our cases emphasize the importance of comprehensive assessment of patients with CMT4D, including pulmonary function tests, vocal cord examination and swallowing assessment, and visual evoked potentials, which may reveal subclinical abnormalities that affect management decisions. While CMT4D remains a rare disease, recognition of its clinical features and genetic basis is important for accurate diagnosis and genetic counseling.

In conclusion, our work expands the genetic spectrum of *NDRG1*-related neuropathy. Continued documentation of detailed clinical and genetic data in rare diseases like CMT4D is essential for advancing our understanding of pathogenic mechanisms and developing targeted therapies.

## Supplemental Material

sj-xlsx-1-jnd-10.1177_22143602261438536 - Supplemental material for Clinical and pathological findings in two Italian siblings of Romani ancestry with charcot-marie-tooth type 4D and review of the current literature Supplemental material, sj-xlsx-1-jnd-10.1177_22143602261438536 for Clinical and pathological findings in two Italian siblings of Romani ancestry with charcot-marie-tooth type 4D and review of the current literature by Elena Abati, Carola Rita Ferrari Aggradi, Stefania Magri, Chiara Pisciotta, Francesca Balistreri, Giacomo Comi, Davide Pareyson, Franco Taroni and Stefania Corti in Journal of Neuromuscular Diseases
